# A GWEP COLLABORATION: IMPLEMENTING THE AWV IN A RURAL PRIMARY CARE CLINIC

**DOI:** 10.1093/geroni/igz038.1446

**Published:** 2019-11-08

**Authors:** Christine McKibbin

**Affiliations:** University of Wyoming, Laramie, Wyoming, United States

## Abstract

This presentation will focus on the collaboration with the Dartmouth GWEP to implement the AWV in a rural primary care clinic. The challenges of practice transformation in busy primary care clinics will be discussed along with lessons learned on a successful GWEP partnership to achieve improved patient outcomes in primary care.

